# Reliability of induced sputum test is greater than that of throat swab test for detecting SARS-CoV-2 in patients with COVID-19: A multi-center cross-sectional study

**DOI:** 10.1080/21505594.2020.1831342

**Published:** 2020-10-19

**Authors:** Tianwen Lai, Fangfei Xiang, Jianfeng Zeng, Yingzi Huang, Liping Jia, Hui Chen, Jiayuan Wu, Jianfeng Xie, Shuna Liu, Wei Deng, Weiqiang Zheng, Yang Huang, Qinfu Zhang, Qingfeng Luo, Fan Mo, Lieming Long, Wuying Zhang, Wenna Chen, Huanqin Han

**Affiliations:** a Department of Pulmonary and Critical Care Medicine, Affiliated Hospital of Guangdong Medical University, Zhanjiang, Guangdong, China; b Infectious Disease Center, Guangzhou Eighth People’s Hospital, Guangzhou Medical University, Guangzhou, Guangdong, China; c Department of Pulmonary Medicine and Tuberculosis, The Third People’s Hospital of Shenzhen, Shenzhen, Guangdong, China; d Department of Critical Care Medicine, Zhongda Hospital, School of Medicine, Southeast University, Nanjing, Jiangsu, China; e Department of Respiratory Medicine, Huanggang Central Hospital, Huanggang, Hubei, China; f Department of Intensive Care Medicine, The First Affiliated Hospital of Soochow University, Suzhou, Jiangsu, China; g Department of Clinical Research, Affiliated Hospital of Guangdong Medical University, Zhanjiang, Guangdong, China; h Infectious Diseases Center, Affiliated Hospital of Guangdong Medical University, Zhanjiang, Guangdong, China

**Keywords:** COVID-19, reliability, induced sputum, throat swab, SARS-CoV-2

## Abstract

We previously reported that sputum induction was more sensitive than throat swabs for the detection of severe acute respiratory syndrome coronavirus 2 (SARS-CoV-2) in two convalescent coronavirus disease 2019 (COVID-19) patients; however, the value and safety of induced sputum testing require further study. We conducted a prospective multi-center cross-sectional study to compare induced sputum to throat swabs for SARS-CoV-2 detection. Confirmed COVID-19 patients from six hospitals in six cities across China who received one or more negative RT-PCR result for SARS-CoV-2 were enrolled, and paired specimens (induced sputum and throat swabs; 56 cases) were assayed. In three paired samples, both the induced sputum and throat swabs were positive for SARS-CoV-2. The positive rate for induced sputum was significantly higher than for throat swabs both overall (28.6% vs 5.4%, respectively; p < 0.01). Patients were divided according to time span from onset of illness to sample collection into the more-than-30-day (n = 26) and less-than-30-day (n = 30) groups. The positive rate for induced sputum was also significantly higher than for throat swabs in the less-than-30-day group (53.3% vs 10.0%, respectively; p < 0.001). For the more-than-30-day group, all paired samples were negative for SARS-CoV-2. Blood oxygen saturation, respiratory rate, and heart rate remained stable during sputum induction and no staff were infected. Because induced sputum is more reliable and has a lower false-negative rate than throat swabs, we believe induced sputum is more useful for the confirmation of COVID-19 and is safer as a criterion for release from quarantine.

## Introduction

In late December 2019, the novel coronavirus disease 2019 (COVID-19) began spreading globally, growing until it eventually resulted in a pandemic. As of 22 September 2020, a total of 30 million confirmed cases have been reported from more than 200 countries, causing nearly 1 million deaths worldwide [1]. To stem this tide, reliable methods for monitoring disease development are needed.

Currently, viral loads are routinely measured to monitor severe viral respiratory tract infections for clinical progression, response to treatment, cure, and relapse. To diagnose COVID-19, specimens are collected from respiratory mucosal surfaces with nasopharyngeal or oropharyngeal swabs [2], both of which have been recommended by the World Health Organization for the detection of severe acute respiratory syndrome coronavirus 2 (SARS-CoV-2). However, a high rate of convalescent COVID-19 cases shows positive reverse transcriptase-polymerase chain reaction (RT-PCR) test results again after discharge [3,4], indicating potential infectivity. Studies have also shown a high false-negative rate for SARS-CoV-2 [5–7], which might prevent patients with COVID-19 from being diagnosed in time or enable convalescent cases to mistakenly meet the criteria for hospital discharge, thus getting released from quarantine, resulting in the spread of disease. Therefore, the appropriate selection of specimens is important for diagnosing COVID-19.

Alveolar lavage fluid (collected via bronchoscopy) is more ideal than nasopharyngeal and oropharyngeal specimens because it is easier to detect SARS-CoV-2 in alveolar lavage fluid; however, because bronchoscopy is invasive and requires maximum protection for patients and medical staff, it should be used sparingly [8]. Sputum might also have a higher detection rate for SARS-CoV-2 than nasopharyngeal or oropharyngeal swabs [9,10]. Sputum is a useful noninvasive method for the detection of SARS-CoV-2 that would aid in the detection of SARS-CoV-2; unfortunately, its use is confined to patients who can produce sputum.

Previous studies have shown that most patients with COVID-19 have symptoms of low sputum production; therefore, it is difficult to obtain sputum in these patients [11,12]. One option is sputum induction by hypertonic saline solution inhalation, which is widely used to study airway secretions in patients with lung diseases such as asthma and chronical obstructive pulmonary disease [13,14]. Indeed, we had previously reported that SARS-CoV-2 RNA was more readily detected in induced sputum than in throat swabs of two convalescent patients with COVID-19 who could not produce sputum [15]. To further assess the potential superiority of induced sputum over throat swabs, we conducted a multi-center cross-sectional study to evaluate the effect and safety of using induced sputum for SARS-CoV-2 detection. To our knowledge, this is the first prospective cross-sectional study to compare the reliability of induced sputum and throat swab tests in patients with COVID-19.

## Methods

### Study design and participants

This multi-center cross-sectional study included inpatient cases from six hospitals in six cities (Affiliated Hospital of Guangdong Medical University; Guangzhou Eighth People’s Hospital, Guangzhou Medical University; The Third People’s Hospital of Shenzhen; Tongji Hospital, Tongji Medical College, Huazhong University of Science and Technology; Huanggang Central Hospital; and Huangshi Hospital of Chinese Medicine). From 13 March to 12 May 2020, all patients who had been diagnosed with COVID-19 according to the guidelines set by the World Health Organization were screened for SARS-CoV-2 infection via real-time reverse transcriptase-polymerase chain reaction (RT-PCR) analysis of throat or nasopharyngeal swabs. Of these patients, all those who received one or more negative RT-PCR result for SARS-CoV-2 were enrolled. The severity of their disease was defined based on Protocols for Diagnosis and Treatment for COVID-19 of China (Trial Version 7) [2].

This study was registered with http://www.chictr.org.cn (ChiCTR-TRC-2000030721).

### Data collection

Clinical characteristics, laboratory results, treatment, and outcome data were extracted from electronic medical records. All data were checked by two physicians (Dr Huanqan Han and Dr Tianwen Lai); a third researcher (Dr Jiayuan Wu) adjudicated any differences in interpretation between the two primary reviewers.

### Specimen collection

Although the United States Centers for Disease Control and Prevention has stated that nasopharyngeal swabs are preferred, throat swabs are more widely used in China. Thus, we did not compare the positive rate for nasopharyngeal swabs.

The procedure for collecting throat swabs and induced sputum was performed before lunch. The subjects were asked to rinse their mouths three times and clean the food residues in the mouth before procedure. The procedure for collecting throat swabs entailed separately swabbing the posterior pharynx and each tonsil with a nylon-flocked swab while avoiding the tongue, then immediately placing the swab into a sterile tube containing 3 mL of viral transport medium. This was promptly followed by induced sputum collection.

Sputum was induced by having each subject inhale a nebulized solution of 3% saline for 20 min. The subjects then spit out saliva, took two deep inspirations of saline, and coughed sputum into a separate cup. Their mouths were rinsed with saline water before sputum induction to minimize oral contamination. Oxygen saturation, respiratory rate, and heart rate were monitored at 1 h and 24 h after sputum induction.

For total RNA extraction and amplification, RT-PCR was performed. A China Food and Drug Administration-approved commercial kit specific for SARS-CoV-2 detection was used and methods similar to those described elsewhere were applied [16].

### Statistical analysis

Continuous variables are presented as the median (interquartile range, IQR) or mean ± standard deviation. Categorical variables are expressed as counts and percentages. We used the Mann–Whitney U test, Wilcoxon signed-rank tests, unpaired *t* test, χ2 test, or Fisher’s exact test to compare differences between induced sputum and throat swabs, as appropriate. Statistical analyses were performed using SPSS 25.0 (SPSS, Inc., IBM, Armonk, NY, USA). A two-sided p-value less than 0.05 was considered statistically significant.

## Results

### Clinical characteristics

A total of 56 cases, including 28 males and 28 females aged 17 to 86 years (median age of 54 years), were enrolled. Among the overall study population, 27 patients (48.2%) had underlying chronic cardio-cerebrovascular or pulmonary diseases. Those and other demographic and clinical characteristics are shown in Table 1.

**Table 1.** Characteristics and symptoms of patients with COVID-19.

Characteristics	All patients (n = 56)
Gender	
Male	28/56 (50.0%)
Female	28/56 (50.0%)
Age (minimum–maximum), years	54 (17–86)
Clinical Classification	
Mild Cases	10/56 (17.9%)
Moderate Cases	38/56 (67.9%)
Severe Cases	8/56 (14.3%)
Underlying chronic cardio-cerebrovascular and pulmonary diseases	27/56 (48.2%)
Hypertension	13/56 (23.2%)
Diabetes	5/56 (8.9%)
Coronary heart disease	4/56 (7.1%)
Cerebrovascular disease	2/56 (3.6%)
Chronic obstructive pulmonary disease	2/56 (3.6%)
Symptoms	
Cough	45/56 (80.4%)
Fever	37/56 (66.1%)
Dyspnea	11/56 (19.6%)
Sputum production	11/56 (19.6%)
Fatigue	10/56 (18.9%)
Diarrhea	9/56 (16.1%)
Sore throat	7/56 (12.5%)
Myalgia	5/56 (8.9%)
Duration of fever (minimum–maximum), days	10 (1–22)

Data are represented as n/N (%).

Upon admission, the severity of COVID-19 was categorized as mild, moderate, and severe in 10, 38, and 8 patients, respectively. Common symptoms were cough, fever, dyspnea, sputum production, fatigue, diarrhea, sore throat, and myalgia. Among them, only 19.6% of patients had sputum production. The median duration of fever was 10 days (range, 1 to 22 days). Lymphocytopenia or leukopenia was present in 26.8% of the patients. Some patients had elevated levels of C-reactive protein, procalcitonin, and lactate dehydrogenase (Table 2). However, laboratory findings (e.g. lymphocytopenia, D-dimer, and procalcitonin) were significantly improved at the time of sputum induction, suggesting that the patients’ condition had improved or stabilized.

**Table 2.** Laboratory findings of patients with COVID-19 upon admission to the hospitals and at the time of sputum induction.

	Upon admission (n = 56)	Time of induced sputum (n = 56)	p value
White blood cell count, × 10⁹ per L	5.35 (3.98–6.85)	5.09 (4.21–6.41)	0.757
< 4.0	15/56 (26.8%)	12/56 (21.4%)	0.508
4.0–10.0	38/56 (68.9%)	41/56 (73.2%)	0.534
> 10.0	3/56 (5.4%)	3/56(5.4%)	1.000
Lymphocyte count, × 10⁹ per L	1.25 (0.78–1.46)	1.48 (1.21–1.95)	0.743
< 0.8	15/56 (26.8%)	4/56 (7.1%)	0.006
≥ 0.8	41/56 (73.2%)	52/56 (92.9)	0.006
D-dimer, mg/L	0.73 (0.39–1.63)	0.50 (0.35–1.22)	0.044
C-reactive protein > 10 mg/L	16/39 (41.0%)	11/39 (28.2%)	0.234
Procalcitonin > 0.05 ng/mL	18/31 (58.1%)	9/31 (29.0%)	0.025
Lactate dehydrogenase, U/L	217.50 (180.50–361.50)	184.50 (164.75–227.25)	0.044
> 250	11/36 (30.6%)	6/36 (16.7%)	0.133
Serum creatinine, μmol/L	64.00 (48.96–78.30)	58.55 (46.67–71.58)	0.075
Alanine aminotransferase, U/L	20.00 (16.26–29.40)	21.50 (14.55–32.35)	0.836
> 40	7/49 (14.3%)	11/49 (22.4%)	0.297
Aspartate aminotransferase, U/L	21.00 (16.95–32.00)	20.00 (16.95–25.50)	0.208
> 40	8/49(16.3%)	9/49 (18.4%)	0.240
Total bilirubin, mmol/L	10.00 (6.25–16.34)	10.00 (6.71–14.02)	0.247
> 17.1	12/49 (24.5%)	6/49 (12.2%)	0.118

Data are represented as median (interquartile range) or n/N (%), where N is the total number of patients with available data. The p values were obtained from χ^2^, Fisher’s exact, or Wilcoxon signed-rank tests.

### Comparisons between induced sputum and throat swab specimens

**Figure 1.**
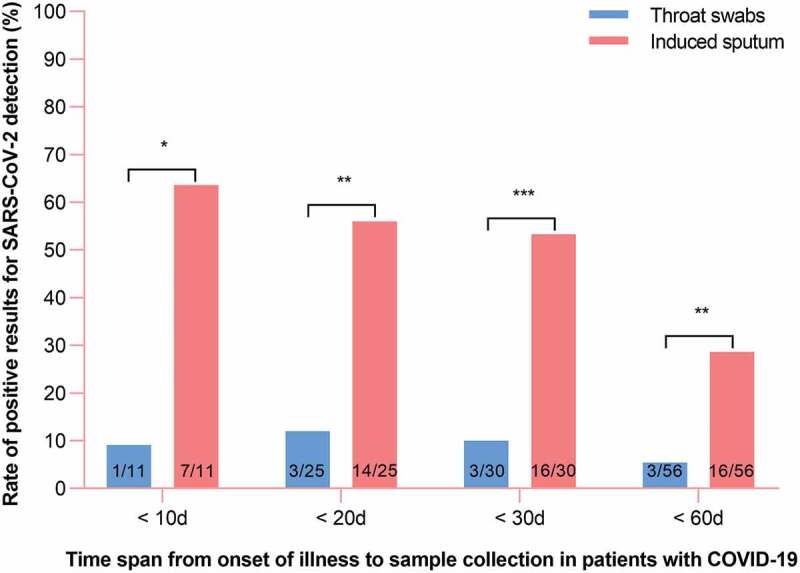
Distribution of RT-PCR results for throat swab and induced sputum specimens in patients with COVID-19.

Fifty-six paired samples of induced sputum and throat swabs were collected. In three paired samples, both the induced sputum and throat swabs were positive for SARS-CoV-2. Overall, induced sputum specimens had a SARS-CoV-2–positive rate that was nearly fivefold that of throat swabs (28.6% vs 5.4%, respectively, p < 0.01; Figure 1).

When time spans from illness onset to sample collection were <10 d, <20 d, <30 d, and <60 d, the positive rates for induced sputum vs throat swab specimens were 63.6% vs 9.1%, 56.0% vs 12.0%, 53.3% vs 10.0%, and 28.6% vs 5.4%, respectively. The numbers in the bars refer to the number of cases. All enrolled patients had at least one negative test for SARS-CoV-2 in respiratory tract specimens. * p < 0.05; ** p < 0.01, *** p < 0.001; p values are from *χ*^2^ or Fisher’s exact test.

The median time from onset of illness to sample collection was 27.0 days (IQR, 12.0 to 48.0). To assess whether the time span from onset of illness to induced sputum collection impacted the rate of positive SARS-CoV-2 detection, patients were divided according to time span from onset of illness to sample collection into the more-than-30-day (n = 26) and less-than-30-day (n = 30) groups. In the less-than-30-day group, the rate of positive SARS-CoV-2 detection was significantly higher for induced sputum than for throat swab specimens (53.3% vs 10.0%, respectively; p < 0.001); However, in the more-than-30-day group, the 26 paired samples were all negative for SARS-CoV-2. These findings suggest that the positive SARS-CoV-2 detection rate for induced sputum decreases with time after the onset of illness; nevertheless, we found that the positive detection rate was significantly higher for induced sputum than for throat swab specimens at all time points (p < 0.05; Figure 1).

### Factors associated with duration of viral shedding

All 56 patients were treated with antiretroviral drugs such as lopinavir/ritonavir, chloroquine, nebulized interferon or arbidol, and all recovered and discharged. The median time from onset of illness to viral shedding was 20.0 days (IQR, 2.0 to 58.0 days). The duration of viral shedding was significantly longer in patients aged 60 years or older as well as in those who suffered underlying chronic cardio-cerebrovascular and/or pulmonary diseases. In contrast, sex, corticosteroid treatment, and obesity had no effect on the duration of viral shedding (Table 3).

**Table 3.** Association between factors and duration of viral shedding.

	Duration of viral shedding		p value
	With factors	Without factors	
Senior (age > 60 years)	24.00 (18.00–35.00); n = 21	17.00 (11.00–26.50); n = 35	0.037
Male	17.50 (12.50–24.00); n = 28	25.00 (12.75–34.25); n = 28	0.076
Underlying diseases*	25.00 (15.75–37.50); n = 20	18.00 (11.00–26.25); n = 36	0.038
Overweight or obese (BMI > 23.9 kg/m^2^)	19.50 (15.25–32.00); n = 14	18.00 (11.00–27.00); n = 31	0.500
Use of glucocorticoids	22.00 (14.00–27.00); n = 18	17.00 (12.00–27.5); n = 38	0.457

*Chronic cardio-cerebrovascular and/or pulmonary diseases. Data are represented as median (interquartile range); n is the number of patients with available data. The p values were obtained from Mann–Whitney U test.

### Adverse events

Given that COVID-19 is an infectious respiratory illness, we assessed the safety of inducing sputum with respect to the medical staff. Nebulization-induced sputum was not collected in the negative pressure wards of any of the six hospitals participating in this study. During the 14-day period after nebulization-induced sputum collection, no medical staff members showed signs of SARS-CoV-2 infection and no nosocomial COVID-19 cases were identified.

We also assessed the safety of patients who underwent induced sputum collection by monitoring their blood oxygen saturation, respiratory rate, heart rate before sputum induction, as well as 1 h and 24 h after the procedure; we also questioned them regarding spontaneously occurring symptoms at those time points. More specifically, we asked patients about the degree (mild, moderate, or severe) to which they perceived chest tightness, shortness of breath, cough, excessive phlegm, sore throat, dry mouth, and other complaints.

**Figure 2.**
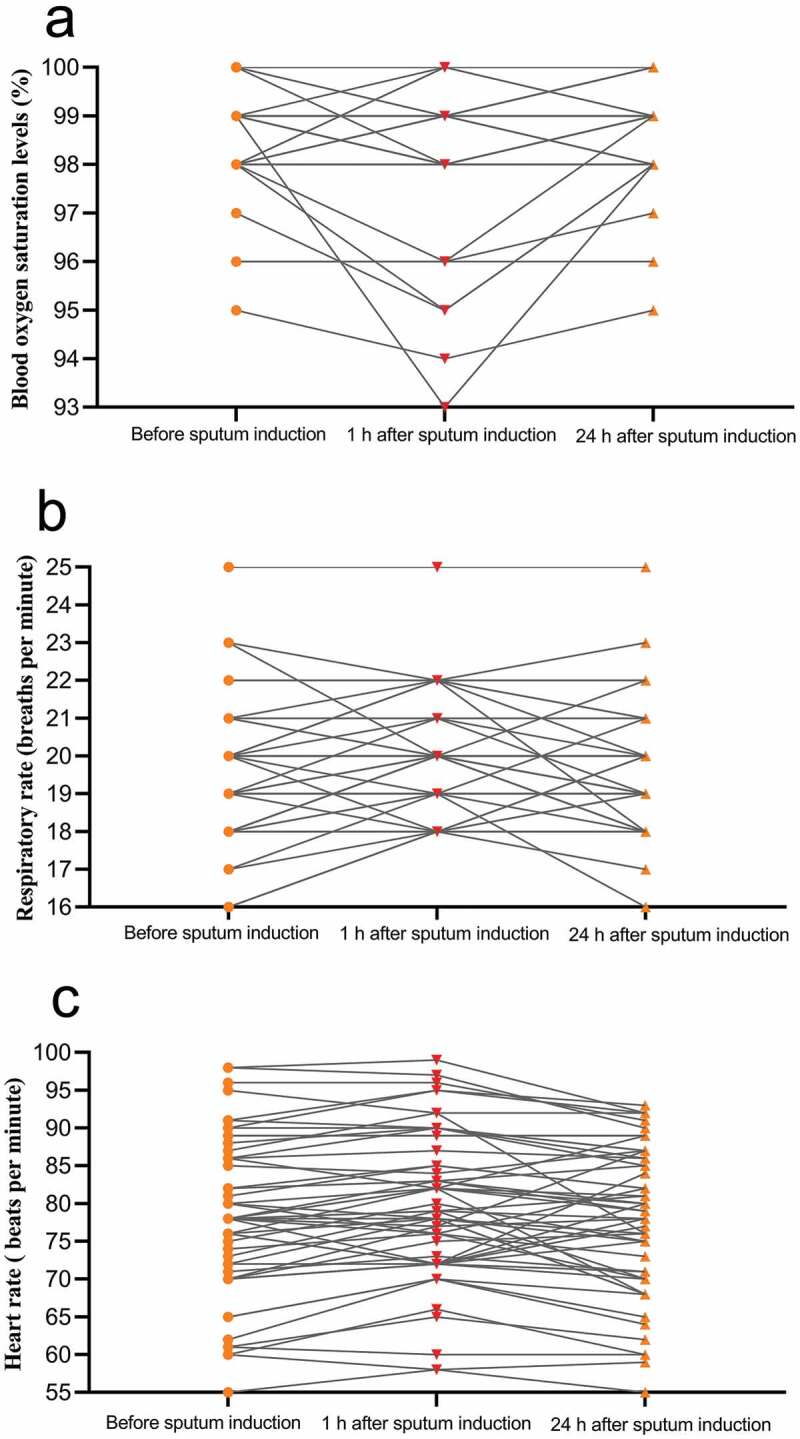
Changes in patients’ vital signs during induced sputum collection.

Symptoms that developed 1 h after induced sputum collection primarily included excessive phlegm (51.8%; 29/56) and mild to moderate cough (50.0%; 28/56); those with a cough had either developed a new mild cough or originally had a mild cough that worsened to a moderate cough. Twenty-four hours after induced sputum collection, only one patient (1/56) had a new mild cough (Table 4). Blood oxygen saturation, respiratory rate, and heart rate were stable during induced sputum collection. At all three time points, all patients had blood oxygen saturation levels exceeding 93%, respiratory rates of 16 to 25 breaths per minutes, and heart rates of 55 to 100 bpm (Figure 2).

**Table 4.** Association between new symptoms and sputum induction.

	Before sputum induction (n = 56)	1 h after sputum induction (n = 56)	New symptoms 1 h after sputum induction	p value^a^	24 h after sputum induction (n = 56)	New symptoms 24 h after sputum induction	p value^b^
Chest tightness	12/56 (21.4%)	14/56 (25.0%)	2/56 (3.6%), mild	0.654	12/56 (21.4%)	0/56 (0)	1.000
Shortness of breath	11/56 (19.6%)	13/56 (23.2%)	2/56 (3.6%), mild	0.654	11/56 (19.6%)	0/56 (0)	1.000
Cough	18/56 (32.1%)	38/56 (67.9%)	28/56 (50.0%)^c^	< 0.001	19/56 (33.9%)	1/56 (1.8%), mild	0.841
Excessive phlegm	0/56 (0)	29/56 (51.8%)	29/56 (51.8%), mild	< 0.001	0/56 (0)	0/56(0)	1.000
Sore throat	1/56 (1.8%)	3/56 (5.4%)	2/56 (3.6%), mild	0.611	1/56 (1.8%)	0/56(0)	1.000
Dry mouth	0/56 (0)	2/56 (3.6%)	2/56 (3.6%), mild	0.476	0/56 (0)	0/56(0)	1.000

Data are represented as n/N (%), where N is the total number of patients. ^a^Before vs 1 h after sputum induction. ^b^Before vs 24 h after sputum induction. ^c^mild, 20/56 (35.7%); from mild to moderate, 8/56 (14.3%). The p values were obtained from χ^2^, Fisher’s exact tests.

(a) One hour after sputum induction, the blood oxygen saturation levels (SpO_2_) of all the patients were all ≥93% (one patient’s SpO_2_ was 93% and one patient’s SpO_2_ was 94%; the remaining patients had SpO_2_ ≥ 95%). Twenty-four hours after sputum induction, the SpO_2_ of all the patients were all ≥95%. (b) At the pre-sputum induction, 1 h post sputum induction, and 24 h post sputum induction time points, all patients had respiratory rates of 16–25 breaths per minutes (c) and heart rates of 55–100 bpm (one patient had a heart rate of <60 bpm).

### Confounding factors leading to high positive rate for induced sputum and high negative rate for throat swabs

We analyzed possible factors that may have led to the high positive rate for induced sputum and low positive rate for throat swabs. For this analysis, patients were divided into the throat swab–positive/induced sputum–negative (i.e. positive-negative) group and the throat swab–negative/induced sputum–negative (i.e. negative-negative) group. Results showed that, compared to patients in the negative-negative group, patients in the positive-negative group were younger, had a shorter time to onset of illness, and were less likely to have underlying diseases (Table 5).

**Table 5.** Association between factors and induced sputum/throat swab results.

	Positive-negative group^a^ (n = 13)	Negative-negative group^b^ (n = 40)	p value
Sex			0.129
Male	4/13 (30.8%)	22/40 (55.0%)	··
Female	9/13 (69.2%)	18/40 (45.0%)	··
Age, years	46.46 ± 14.04	57.45 ± 19.91	0.036
BMI, kg/m^2^	23.50 ± 3.78	23.95 ± 3.32	0.700
Time to onset of illness, days	12 (8–14)	40 (18–51)	0.000
Fever	6/13 (46.2%)	24/40 (60.0%)	0.539
Cough	10/13 (76.9%)	33/40 (82.5%)	0.655
Fever duration, days	1 (0–7)	4.5 (0–11.25)	0.320
Underlying diseases^c^	1/13 (7.7%)	19/40 (47.5%)	0.025
Severe cases	3/13 (23.1%)	5/40 (12.5%)	0.632
Imaging of pneumonia	11/13 (84.6%)	33/40 (82.5%)	1.000

^a^Throat swab–positive/induced sputum–negative group. ^b^Throat swab–negative/induced sputum–negative group. ^c^Chronic cardio-cerebrovascular or pulmonary diseases. Data are represented as median (interquartile range), mean ± standard deviation or n/N (%). The p values were obtained from χ^2^, Fisher’s exact, unpaired *t*, or Mann–Whitney U tests.

## Discussion

SARS-CoV-2 detection rate might be higher for sputum samples than for nasopharyngeal or throat swabs [9,10]. However, in order to test sputum, patients must produce sputum. In our study, approximately 80% of patients with COVID-19 had no symptoms of sputum production, a situation that has been similarly reported by others [11,12]. Induced sputum is representative of viral conditions in the lower respiratory tract. It is widely used to study airway secretions in patients with lung diseases such as asthma and chronical obstructive pulmonary disease and is a useful noninvasive method for detecting SARS-CoV-2 [13,14]. Thus, it is vital to assess the clinical value and safety of induced sputum in patients with COVID-19 who are unable to readily produce sputum.

The current study enrolled patients who presented themselves with mild to severe COVID-19 during various stages of the disease, including the prodromal, apparent manifestation, and convalescent periods. Therefore, the enrolled patients were fairly representative although the point of stabilization or recovery tended to occur during the sputum induction period.

In the present study, pairs of induced sputum and throat swab specimens were obtained from 56 patients with COVID-19 who had at least one negative RT-PCR result for SARS-CoV-2. During the study, RT-PCR assays revealed that overall, the positive SARS-CoV-2 rates were significantly higher for induced sputum than for throat swab specimens (28.6% vs 5.4%, respectively); the differences were especially apparent when the time from onset of illness to sample collection was less than 30 days (53.3% vs 10.0%, respectively). These findings were consistent with the results of our previous study [15]. Collectively, our studies demonstrate that a negative throat swab does not indicate the absence of respiratory SARS-CoV-2 infection. Thus, it may be necessary to reevaluate the suitability of discharge criteria, which currently require two consecutive negative SARS-CoV-2 tests of respiratory samples at least one day apart. We suggest that the discharge criteria include at least one induced sputum test.

The hypertonic saline used for sputum induction is known to cause adverse reactions owing to stimulation of the respiratory tract; these reactions tend to be mild and well tolerated. Nevertheless, we carefully evaluated the safety of inducing sputum in patients with COVID-19. Our research showed that the primary effects associated with sputum induction were mild cough and sputum production, but inducing cough and collecting “high-quality” sputum from deep within the trachea were the goal of the procedure; moreover, these symptoms were relieved within a short period of time. The number of other obvious adverse effects was insignificant, and objective vital signs (blood oxygen, breathing, heart rate) remained stable after sputum induction. Taken together, our results suggest that inducing sputum is safe for patients with COVID-19.

Some scholars argue that nebulization in the general ward could aerosolize SARS-CoV-2, thereby increasing the risk of employee exposure. It is not possible to promote the construction of negative pressure wards in areas where COVID-19 is prevalent or experiencing outbreaks owing to the high standards required for constructing and maintaining them. However, negative pressure wards may not be necessary for sputum induction, as the risk of nebulization-associated SAR-CoV-2 aerosolization has not been confirmed by the current prevention and control practices for COVID-19. In China, nebulized interferon is commonly used in many hospitals for the prevention and treatment of COVID-19, but these hospitals have not reported an increased incidence of SARS-CoV-2 infections in their medical staff. In our study, we nebulized patients in the general ward, but no medical staff was infected with SARS-CoV-2 during that time. Therefore, we believe that the sputum induction process does not pose any risk of infection to medical personnel.

We explored possible confounding factors that may have contributed to the high positive detection rate for SARS-CoV-2 in induced sputum specimens. Results suggest that young patients and patients with a relatively short course of disease were more prone to having positive-negative results than old patients with a relatively long course of the disease. This possibility was based only on our assessment of the data obtained in our study. However, the underlying reason may be found within the characteristics of COVID-19 itself and the quality of sputum specimens. First, angiotensin-converting enzyme 2 receptors that reside in the alveolar cells of the lower respiratory tract may be the receptors for SARS-CoV-2 [17]. Therefore, the virus is more likely to attack the lower respiratory tract than the pharynx. It follows that because induced sputum samples come from the lower respiratory tract, induced sputum samples are more likely than pharyngeal (i.e. throat swab) samples to produce positive SARS-CoV-2 results. Second, quality control during throat swab collection cannot be guaranteed. Some medical staff may not have collected high-quality specimens owing to their fear of exposure; there are no such problems with induced sputum.

This study has some limitations. First, nucleic acid kits were not uniform among the hospitals. However, we ensured that the testing laboratories were qualified and that the kits were approved by the China Food and Drug Administration; moreover, each paired sample was collected using the same kit. Second, owing to the sharp reduction in COVID-19 cases over the past 3 months in China, we did not include a large number of cases; instead, we only selected more representative cities, hospitals, and patients for this study. We believe that the current samples are sufficient to illustrate the problem with throat swab testing.

In fact, more and more studies now show that the specimens used for pathogenic diagnosis of COVID-19 are not limited to respiratory specimens such as sputum or throat swabs. Saliva is a good example. Its collection is fast, easy, inexpensive, and noninvasive and can be used to identify various oral and systemic conditions [18,19]. In the field of infectious diseases, saliva testing already include viral infections such as dengue, severe acute respiratory syndrome (SARS) and Middle East respiratory syndrome (MERS). [20] Furthermore, recent studies have shown that saliva has certain advantages in diagnosing COVID-19 [21,22]. Therefore, it is extremely important to choose appropriate samples such as induced sputum and saliva, to detect SARS-Cov-2 in patients with COVID-19 according to the conditions of patients and hospital.

## Conclusions

We found that throat swab tests had a higher false-negative rate than induced sputum tests, and induced sputum tests were more sensitive than throat swab tests. In addition, induced sputum collection is a simple, noninvasive, and safe procedure. Therefore, for imaging or epidemiologically suspected cases of COVID-19 in which throat swabs are negative or for difficult-to-diagnose COVID-19 cases, induced sputum may be useful for the confirmation of COVID-19. For convalescent patients with COVID-19, negative-induced sputum should be used as a criterion for discharge from the hospital and release from quarantine.
